# Antireflective Transparent Conductive Oxide Film Based on a Tapered Porous Nanostructure

**DOI:** 10.3390/mi11020206

**Published:** 2020-02-17

**Authors:** Kiwoon Choi, Jaehoon Jung, Jongyoung Kim, Joonho Lee, Han Sup Lee, Il-Suk Kang

**Affiliations:** 1 Next E&M Research Institute, Environmental Research Center, 410 Jeongseojin-ro, Seo-gu, Incheon 22689, Korea; 2 Department of Chemical Engineering, Inha University, 100 Inha-ro, Nam-gu, Incheon 22212, Korea; 3 National Nanofab Center, Korea Advanced Institute of Science and Technology, 291 Daehak-ro, Yuseong-gu, Daejeon 34141, Korea

**Keywords:** antireflective film, transparent conductive oxide, nanostructure

## Abstract

A new architecture for antireflection (AR) has been developed to break the trade-off between the optical transmittance and the electrical conduction impeding the performance of transparent conductive oxide (TCO) films. The tapered porous nanostructure with a complex continuous refractive index effectively eliminates reflections from the interfaces between air and the TCO and TCO and the substrate. Compared to the conventional TCO film, the AR TCO film exhibited the same electrical conduction, with an average transmittance of 88.7% in the 400–800 nm range, a 10.3% increase. The new AR TCO film is expected to improve the performance of various optoelectronic devices.

## 1. Introduction

A transparent conductive oxide (TCO) film, applied as a transparent electrode, is an essential component of various optoelectronic devices such as solar cells, display panels, and light-emitting devices [1,2,3,4,5,6,7]. TCO films are required to have both sufficient electrical conduction and good optical transmittance. However, there is a trade-off between these parameters, thus presenting a major challenge in the development of TCO films. In this study we propose a new architecture for antireflection (AR) employing a tapered porous nanostructure with a complex continuous refractive index and demonstrate its potential for breaking this trade-off.

**Figure 1:**
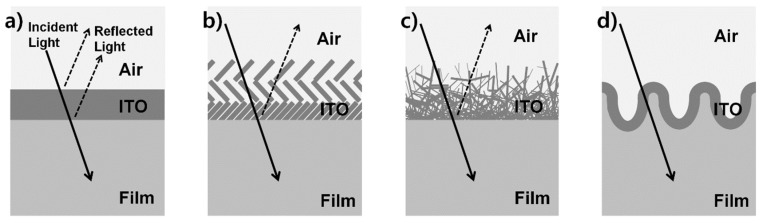
Schematic diagrams showing the structures of the conventional λ/4 antireflection (AR) coating transparent conductive oxide (ITO) film (**a**) and the nano-porous ITO film (**b**) and highly branched ITO nanowire film (**c**) and the new structure for an ideal complex continuous refractive index (CCRI) ITO film designed in this work (**d**).

Among various TCO materials indium tin oxide (ITO) has been extensively used in a wide range of applications due to its high transmittance and low sheet resistance [8,9,10]. Figure 1a shows a schematic diagram of the conventional ITO film structure having three interfaces (air-ITO, ITO-substrate, and substrate-air). Reflection occurs at the interfaces between two media having a different refractive index and this causes decreased transmittance and contrast, resulting in performance degradation of optoelectronic devices based on ITO films.

Various AR technologies such as a *λ*/4 AR coating, multilayer coating, and antireflective structure (ARS) have been developed to minimize the reflection from the air-substrate [11,12,13,14]. However, most reflection occurs from the air-ITO and ITO-substrate interfaces rather than from the air-substrate interface, and thus it is necessary to develop a new AR technology to reduce the reflection from all three interfaces. A typical approach to control the reflection from the interface between two media having different refractive indices is to insert a layer with a continuous refractive index change from one medium to another [12]. By optimizing the optical impedance matching with the continuous refractive index layer it is theoretically possible to realize broadband as well as omnidirectional AR performance.

With the recent progress of ITO deposition technologies, nanostructured continuous refractive index ITO film methods such as nano-porous ITO [15] and highly branched ITO nanowire methods [16,17] have been developed. When the nanostructured continuous refractive index ITO film is used as a topmost contact electrode in light-emitting devices, its efficiency can be significantly enhanced. Figure 1b,c show schematic diagrams of AR ITO films based on nanostructured continuous refractive index layers [15,16,17]. As these layers consist of two components, air and ITO, they may provide a less abrupt refractive index change at the interfaces from air to ITO. Nevertheless, owing to the refractive index difference between the ITO and the substrate, strong reflection from the ITO-substrate still cannot be avoided. Furthermore, owing to the high roughness of the nanostructured ITO surface, delicate patterning with photolithography and etching cannot be easily carried out.

Figure 1d shows a schematic diagram of the new structure for an ideal complex continuous refractive index (CCRI) ITO film. As the film consisting of three components, air, ITO, and substrate, offers a continuous refractive index change from the top air layer to the bottom substrate, reflection from the air-ITO and ITO-substrate can be effectively reduced. If the surface takes the form of smooth curves and rounded edges, lamination on the ITO layer and patterning of the layer are expected to be easily carried out.

It has been reported that the reflection from the air-substrate can be successfully removed by forming either tapered nipples or tapered pores (negative ARS, N-ARS) on the substrate [18,19]. Monolithic tapered structures of subwavelength size induce a continuous material fraction change from air to the substrate, resulting in very low surface reflection. In the case of a monolithic N-ARS film, its high mechanical stability enables it to be mass-produced through a roll-to-roll process [19]. By coating the ITO layer evenly on the surface of N-ARS film, it is possible to simply fabricate CCRI ITO film for the industrial production.

## 2. Materials and Methods

**Figure 2:**
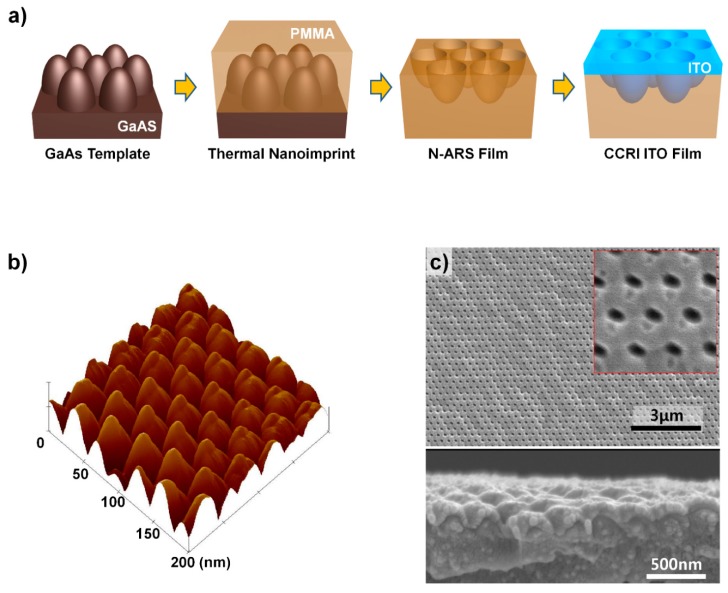
(**a**) Fabrication process of CCRI ITO film. (**b**) AFM height image of GaAs template having paraboloid-shaped nipples. (**c**) Top and cross-sectional SEM images of CCRI ITO film. An enlarged image is provided in the inset.

Figure 2a shows the fabrication process of the CCTI ITO film. In order to produce the CCRI ITO film, a GaAs template with paraboloidal nipples of subwavelength size in a hexagonal array was initially prepared, as shown in Figure 2b [20]. Using a thermal nanoimprint process, tapered pores of approximately 200 nm depth with a 290 nm lattice period were fabricated on the PMMA film surface [19]. Afterward, ITO was coated on the surface of an N-ARS film via sputtering. Thereafter, no additional heat treatment was performed due to the low thermal properties of the PMMA film (not only CCRI but all ITO films on PMMA in this study). Figure 2c shows that while ITO somewhat depended on the position of the nipples, ITO was coated continuously on the surface of the N-ARS film. It is also noted that the thickness was smaller than 50 nm on the flat PMMA surface without ARS coated simultaneously, because the N-ARS increases the actual surface area by more than 1.8 times.

To evaluate electrical conduction, the sheet resistance of flat ITO and CCRI ITO films has been measured with a 4-point probe method [21]. The sheet resistance of the CCRI ITO film (142.0 Ω/sq) is more than 1.8 times that of the flat ITO film (77.2 Ω/sq). If the actual surface area is considered to be more than 1.8 times greater, the sheet resistance of the CCRI ITO film can be considered reasonable. This confirms that ITO covered the surface of N-ARS film without any gaps. Also, when ITO was deposited twice on the surface of the N-ARS film, the sheet resistance of CCRI ITO film was 75.2 Ω/sq, which is comparable to the resistance of the flat ITO film of 50 nm thickness.

## 3. Results and Discussion

**Figure 3:**
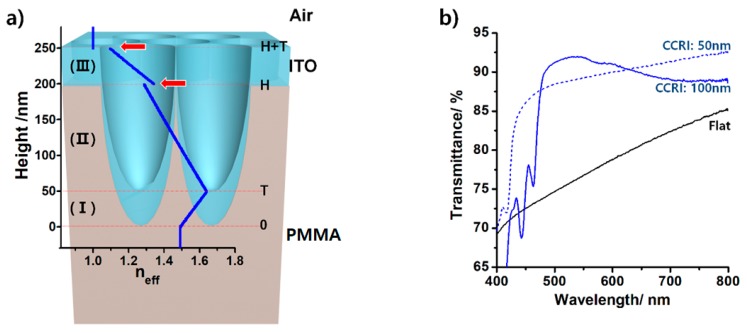
(**a**) Effective refractive index profile (*n_eff_*, blue line) of CCRI-ITO film along the film thickness direction. The refractive index discontinuities are indicated by the red arrows. (**b**) Optical transmittance over the visible wavelength range for CCRI-ITO films with the same (CCRI1, blue dotted line) and twofold higher (CCRI2, blue solid line) deposition time as the 50 nm thick flat ITO film (transmittance indicated by black line).

The reflection at the interfaces in the CCRI ITO film is governed by the refractive index profile along the film thickness direction, from the top air to the bottom substrate. The effective refractive index profiles of the corresponding structure to the CCRI ITO film are shown in Figure 3a. Assuming that the ARS is the subwavelength structure, that is, assuming the lattice period (*d*) of ARS is sufficiently smaller than the wavelength of light in the medium (*d* < *λ*/*n*; at a low visible wavelength range, *d* > *λ*/*n*), according to the Bruggeman effective medium model, the effective refractive index (*n_eff_*) of a plane of any specific height (*z*) in the CCRI ITO film is given as follows [22,23]

(1) $$f_{PMMA}\frac{n_{PMMA}^{2}-n_{eff}^{2}}{n_{PMMA}^{2}+2n_{eff}^{2}}+f_{ITO}\frac{n_{ITO}^{2}-n_{eff}^{2}}{n_{ITO}^{2}+2n_{eff}^{2}}+f_{air}\frac{n_{air}^{2}-n_{eff}^{2}}{n_{air}^{2}+2n_{eff}^{2}}=0$$

Here, *f* is the material fraction at *z*. *n_PMMA_*, *n_ITO_*, and *n_air_* are 1.49, 2.19, and 1, respectively. As noted in Figure 3a, the *z*-axis is perpendicular to the PMMA film surface and the tips of pores in the N-ARS are at *z* = 0. H and T are the height of ARS and the thickness of the ITO layer, respectively. On the basis of its components, the CCRI ITO film can be divided into three different sections: section I (0 ≤ *z* < T) consisting of PMMA and ITO, section II (T ≤ *z* < H) consisting of PMMA, ITO, and air, and section III (H ≤ *z* ≤ H+T) consisting of ITO and air. In section I, *f_PMMA_*(*z*) + *f_ITO_*(*z*) = 1 and *f_air_*(*z*) = 0; in section II, *f_PMMA_*(*z*) + *f_ITO_*(*z*) + *f_air_*(*z*) = 1; and in section III, *f_ITO_*(*z*) + *f_air_*(*z*) = 1 and *f_PMMA_*(*z*) = 0. Using these material fraction relations and equation (1), the effective refractive index profile of the CCRI ITO film with H = 200 nm and T = 50 nm has been calculated, as shown in Figure 3a. Owing to the tapered shape of ARS, it is possible to significantly reduce the abrupt refractive index change from air to the end of ARS. However, there are two refractive index discontinuities, at *z* = H and H + T, which is inevitable because there are abrupt changes of the material fraction.

As the ITO films are generally evaluated by the transmittance, the AR property of CCRI ITO films was confirmed by measuring the transmittance. The transmittance (normal incidence) of visible light through CCRI ITO films of the same (CCRI1, blue dotted line) and twofold greater (CCRI2, blue solid line) deposition time as employed for the 50 nm thick flat ITO film has been measured and the results are shown in Figure 3b. Compared to the flat ITO film (black line), the transmittance of both CCRI ITO films is significantly improved. The transmittance of the CCRI2 ITO film with similar electrical conduction to the flat ITO film varies from 69.3% to 92.8%. The average transmittance is 88.7%, which is 10.3% higher than that of the flat ITO film. The sudden decrease of transmittance over a range of 400–450 nm for the CCRI ITO films is ascribed to a diffraction phenomenon occurring at a low wavelength range where *d* > *λ*/*n*. Since various factors such as *d*, H, T, and the nipple diameter are precisely tunable, further enhancement of the optical transmittance of the CCRI ITO film (to the UV region of 400 nm or less) can be expected.

## 4. Conclusions

In summary, a new architecture has been designed to efficiently eliminate the reflection at the interfaces of multilayer-based optical systems. A complex continuous refractive index structured ITO film that alleviates the sudden refractive index changes in the existing ITO films was successfully fabricated. The obtained film showed significantly enhanced optical transmittance while maintaining high electrical conduction. Furthermore, delicate processes can subsequently be performed, and thus the AR ITO film is expected to significantly enhance the optical performance of various optoelectronic devices.
